# Parkinsonism in Alzheimer's disease without Lewy bodies in association with nigral neuron loss: A data‐driven clinicopathologic study

**DOI:** 10.1002/alz.14628

**Published:** 2025-03-05

**Authors:** Daisuke Ono, Hiroaki Sekiya, Alexia R. Maier, Melissa E. Murray, Shunsuke Koga, Dennis W. Dickson

**Affiliations:** ^1^ Department of Neuroscience Mayo Clinic Jacksonville Florida USA; ^2^ Department of Laboratory Medicine and Pathology Mayo Clinic Jacksonville Florida USA; ^3^ Department of Pathology and Laboratory Medicine Hospital of the University of Pennsylvania Philadelphia Pennsylvania USA

**Keywords:** Alzheimer's disease, artificial intelligence, ChatGPT, digital pathology, machine learning, TAR DNA‐binding protein 43, whole slide imaging

## Abstract

**INTRODUCTION:**

Parkinsonism in patients with Alzheimer's disease (AD) is often attributed to Lewy‐related pathology, given its high comorbidity. In the era of anti‐amyloid therapy, recognizing parkinsonism caused by AD pathology is needed to optimize the treatment.

**METHODS:**

This study aimed to quantitatively characterize parkinsonism and nigral neuropathology in AD without Lewy bodies (LB). Nigral neurons were counted automatically. Fine‐tuned ChatGPT collected structured clinical data.

**RESULTS:**

Among 635 AD patients without LB, 62 (9.7%) presented parkinsonism, which correlated with reduced nigral neuron density (*p* < 0.01). Tau burden did not explain the nigral neuronal loss. TAR DNA‐binding protein 43 (TDP‐43) pathology correlated with reduced nigral pigmented neuron density (*p* = 0.03).

**DISCUSSION:**

Our findings suggest that parkinsonism in AD without LB is related to nigral neuronal loss in association with TDP‐43 pathology. Recognition of parkinsonism in AD without LB is crucial for appropriate therapy.

**Highlights:**

- One in 10 Alzheimer's disease (AD) patients without Lewy bodies had parkinsonism.
- Parkinsonism in AD was correlated with reduced nigral neuron density.
- TAR DNA‐binding protein 43 pathology was associated with nigral degeneration in AD.
- AD should be included in the differential diagnosis of dementia with parkinsonism.

## 1 BACKGROUND

Parkinsonism is a syndrome characterized by bradykinesia, rigidity, and rest tremor. These symptoms are caused by dysfunction of the nigrostriatal system, which typically occurs in patients with Lewy body disease (LBD). Given that 22% to 33% of Alzheimer's disease (AD) cases have concomitant LBD,^1^, ^2^, ^3^ parkinsonism observed in patients with dementia has often been attributed to comorbid LBD. However, several *post mortem* studies have reported parkinsonism in AD in the absence of Lewy bodies (LB).^4^, ^5^ Because these studies included AD with LB and focused more on the importance of comorbid LB in the substantia nigra (SN), the clinical significance of parkinsonism in AD without LB tends to be underestimated. Actually, diagnostic criteria and recent reviews on AD rarely discuss parkinsonism in AD without LB.^6^, ^7^, ^8^, ^9^ However, with the advent of disease‐modifying therapies, a detailed understanding of parkinsonism caused by AD pathology has become important to appropriately select patients for anti‐amyloid therapies.^9^, ^10^, ^11^, ^12^, ^13^

Neuropathologically, parkinsonism is associated with a reduced number of neurons in the SN in Parkinson's disease (PD) and elderly individuals,^14^, ^15^, ^16^, ^17^ but the association in AD without LB has not been elucidated, presumably due to methodological limitations. In previous studies, nigral neurons were manually counted^14^, ^15^, ^16^ or semi‐quantitatively assessed.^4^, ^5^ Manual counting is tedious and time consuming and may not be applicable to large‐scale datasets. Semi‐quantitative scoring, which is typically a 4‐point scale evaluated by neuropathologists, may be inferior to neuron counting in terms of statistical power. Inter‐rater variability and reproducibility have been limitations of neuropathologic evaluation.^18^ Automation of neuron detection is key to ensuring the quantity and quality of data. Our previous machine learning studies quantified neuropathology by detecting tau‐positive lesions in brain specimens, myelinated nerve fibers in peripheral nerves, and myofibers in biopsied specimens.^19^, ^20^, ^21^ The current study sought to expand our framework to counting neurons in the SN to reveal the relationship between parkinsonism and nigral pathology in AD.

**RESEARCH IN CONTEXT**

1. **Systematic review**: The authors conducted a large‐scale clinicopathologic study to evaluate parkinsonism in autopsy‐confirmed Alzheimer's disease (AD) without Lewy bodies using dedicated machine learning programs that automatically count neurons in the substantia nigra and collect structured clinical data.
2. **Interpretation**: Our results show that a subset of AD patients has parkinsonism with nigral neuronal loss that is not associated with Lewy body pathology, but is rather with nigral neuronal loss and TAR DNA‐binding protein 43 pathology. AD patients with parkinsonism tend to be misdiagnosed with other parkinsonian disorders such as Parkinson's disease and corticobasal syndrome. AD should be included in the differential diagnosis of dementia and parkinsonism.
3. **Future directions**: Recent clinical trials of anti‐amyloid therapy may have excluded AD patients based on the combination of dementia and parkinsonism. Our findings caution the possibility of missing potential treatment opportunities in these patients. Appropriate patient selection strategies for disease‐modifying therapy in neurodegenerative diseases should be developed.

For large‐scale clinicopathologic studies, clinical data abstraction from medical records is also a limiting factor, requiring expert resources and raising concerns about inter‐rater variability.^22^, ^23^ Recently, ChatGPT and other large language models have been explored to automate the clinical data collection.^24^, ^25^, ^26^, ^27^, ^28^, ^29^ However, these models have been used for relatively simple tasks, and the reliability of their outcomes has been difficult to interpret. To achieve better outcomes, the development of more comprehensive and interpretable methods for clinical data abstraction is necessary.

The current study aimed to quantitatively characterize the relationship between parkinsonism and nigral neuropathology in AD without LB. To achieve this, we implemented two artificial intelligence pipelines: YOLOv8 for neuron detection and fine‐tuned ChatGPT for clinical data abstraction. Using this data‐driven approach will enhance our understanding of the role of nigral degeneration in AD‐related parkinsonism.

## 2 METHODS

### 2.1 Subjects

**FIGURE 1:**
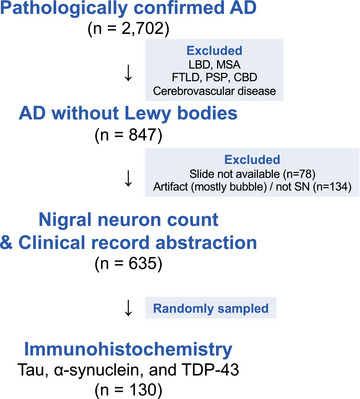
Patients’ selection flow. Among 2702 patients with neuropathologically confirmed Alzheimer's disease (AD), those with Lewy body disease (LBD), multiple system atrophy (MSA), frontotemporal lobar degeneration (FTLD), progressive supranuclear palsy (PSP), corticobasal degeneration (CBD), and cerebrovascular disease are excluded. Cases with limbic‐predominant age‐related TAR DNA‐binding protein 43 (TDP‐43) encephalopathy (LATE) were not excluded, resulting in 847 AD cases without Lewy bodies. After exclusion of unavailable slides and artifacts, 635 cases are evaluated with nigral neuron count and symptom abstraction, of which 130 cases are randomly selected for immunohistochemistry, irrespective of parkinsonism evaluations.

In this retrospective observational study, 2702 patients with neuropathologic diagnosis of AD were included from 9640 donors at the Mayo Clinic Brain Bank accessioned between 1998 and 2022 (Figure 1). Patients with other neurodegenerative diseases, such as LBD (including amygdala‐predominant LBD), multiple system atrophy, frontotemporal lobar degeneration (FTLD), progressive supranuclear palsy (PSP), corticobasal degeneration (CBD), and those with significant cerebrovascular disease were excluded. Patients with limbic‐predominant age‐related TAR DNA‐binding protein 43 (TDP‐43) encephalopathy (LATE) were not excluded, given that one aim of this study was to evaluate TDP‐43 pathology in AD without LB. Available midbrain sections with hematoxylin and eosin (H&E) staining were scanned, and sections with severe artifacts in the SN were excluded from subsequent clinicopathologic evaluation.

### 2.2 Neuropathologic evaluation

Formalin‐fixed brains were sampled using a standardized method by a board‐certificated neuropathologist (D.W.D.).^19^ Paraffin‐embedded 5 µm thick sections mounted on glass slides were stained with H&E and thioflavin S. Immunohistochemistry was performed on midbrain sections for phosphorylated tau (CP13; mouse monoclonal, 1:1000; from the late Dr. Peter Davies, Feinstein Institute for Medical Research), phosphorylated TDP‐43 (p409/410; mouse monoclonal, 1:5000; CosmoBio USA), and phosphorylated α‐synuclein (pS129; rabbit monoclonal, 1:40,000 Abcam Limited) using an immunohistochemistry autostainer (Thermo Fisher Scientific), with Dako EnVisionTM + reagents (Agilent Technologies), and 3, 3‐diaminobenzidine (DAB) as the chromogen. These antibodies have been widely applied to human brain samples, including the substantia nigra, with no reported cross‐reactivity with pigment.^30^, ^31^ Immunostained slides were counterstained with hematoxylin and coverslipped.

Neuropathologic diagnoses were made by a neuropathologist (D.W.D.). Neurofibrillary tangles (NFTs) and senile plaques were quantified by thioflavin S fluorescence microscopy in association cortices (frontal, temporal, and parietal), primary cortices (visual and motor), hippocampus (CA1, CA4, and subiculum) and adjacent cortex, amygdala, basal ganglia, and cerebellum. Braak NFT stage (0–VI) and Thal amyloid phase (0–5) were determined based on the distribution and counts of NFTs and senile plaques, respectively.^32^, ^33^ The pathologic diagnosis of AD and its subtyping was made according to previous publications.^34^, ^35^, ^36^ Presence of TDP‐43 pathology in the SN was evaluated by another neuropathologist (S.K.) who was blinded to clinicopathologic information.

### 2.3 Digital pathology

Whole slide images (WSIs) were obtained by scanning at x20 magnification on the ScanScopeXT (Aperio Technologies), saved in svs format, and analyzed on Qupath 0.4.3.^37^ After normalization by estimation of staining vectors, the SN was manually segmented by an investigator (D.O.). In this study, the SN was defined as a region surrounded by the midbrain tegmentum and cerebral peduncle at the level of the oculomotor nerve roots.^15^ In the DAB channel, pigments in the SN are characterized by a less intense signal than NFTs and a larger area than neuropil threads. Therefore, we segmented tau‐immunoreactive objects, excluding pigments by taking the following steps (Figure S1 in supporting information). (1) The low‐intensity threshold with a cutoff of 0.3 segmented both tau‐immunoreactive objects and pigments. (2) Larger objects such as pigments and NFTs were excluded at a cutoff of 50 µm^2^. (3) NFTs were detected by the high‐intensity threshold filter at a cutoff of 0.8 and included again. When the third step was completed, we measured the tau immunoreactive area and calculated the percentage area occupied in the whole SN.

### 2.4 Machine learning for nigral neuron detection

**FIGURE 2:**
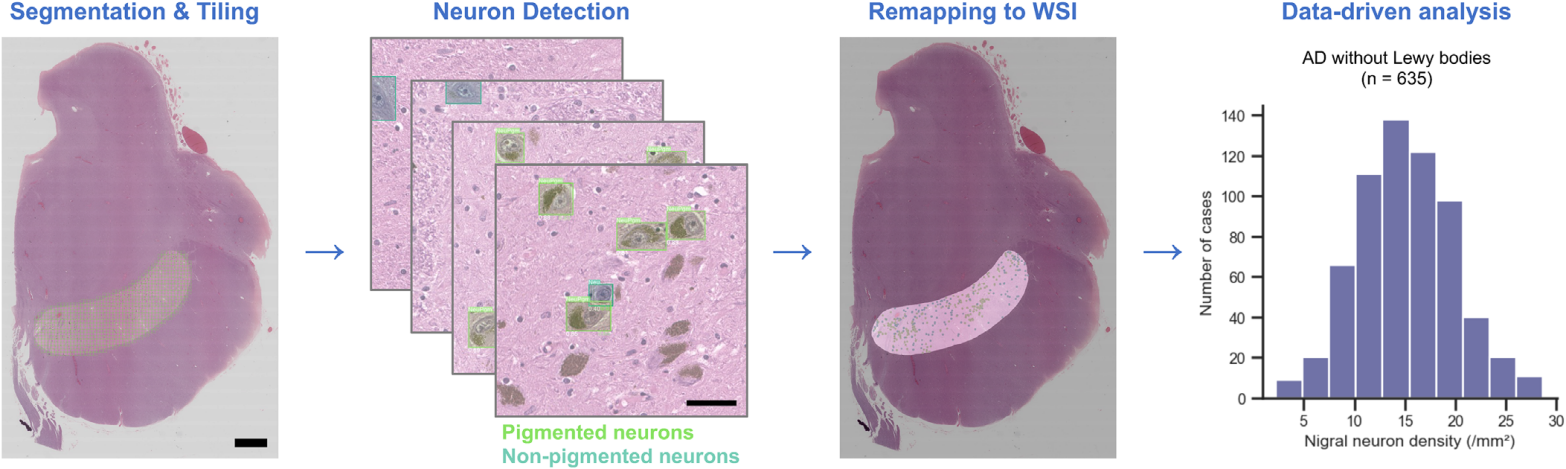
Automated nigral neuron detection on whole slide images (WSIs). The substantia nigra on an hematoxylin & eosin–stained WSI is segmented manually and tiled (leftmost panel). Pigmented neurons (light green) and non‐pigmented neurons (cyan) are detected using the fine‐tuned YOLOv8 model with an mAP50‐95 of 0.651 (second from left). The detected neurons are then remapped to the WSI (second from right). A data‐driven analysis is performed on 635 Alzheimer's disease (AD) cases without other major neurodegenerative diseases, exemplified by a histogram depicting the distribution of nigral neuron density (rightmost panel). Scale bars: 2 mm for the WSI and 50 µm for the tiled image.

We randomly selected from 232 H&E‐stained slides of the midbrain with varying degrees of neurodegeneration in the SN, including 140 cases of LBD, 61 AD, 5 pathological aging, 5 FTLD, 3 PSP, and 3 primary age‐related tauopathy. The SN was identified and segmented by the same definition as that applied in the tau segmentation process by D.O. (Figure 2). From these 232 segmented SNs, 3648 square tiles of 500 pixels were randomly cropped. Neurons with a visible cell body and a nucleolus on the 3648 tiles were identified and labeled as pigmented or non‐pigmented neurons by two investigators (D.O. and A.R.M.) under the supervision of a neuropathologist (D.W.D.).^14^ To adequately discriminate neurons with a nucleolus from those without it, we also added labels for pigmented or non‐pigmented neurons without nucleoli, which were used only for the development of the machine learning model, but not for the subsequent neuron count analysis. The annotated images were randomly assigned to the training, validation, and test datasets in a ratio of 0.70:0.15:0.15. Using the test and validation datasets, an object detection model, yolov8m‐det from YOLOv8.0.99 (https://github.com/ultralytics/ultralytics), was fine‐tuned for 100 epochs with a batch size of 16. Model performance for the test dataset was evaluated as the mean average precision (mAP, IoU = 0.50:0.95), which ranges from 0 to 1, with values closer to 1 indicating better performance.

For neuron detection inference, 500‐pixel square tiles with 50‐pixel overlapping edges were created from a segmented SN image. The fine‐tuned model predicted neurons on the tiled images, which were remapped to the WSI. Neurons truncated by the tile border were excluded as described previously.^20^ If a neuron had multiple labels, the label with the highest confidence score was selected. The model was developed using Python 3.10.12, PyTorch 2.1.0+cu121, and related packages on a cloud computing platform, Google Colab (GPU: A100‐SXM4‐40GB, NVIDIA).

### 2.5 ChatGPT fine‐tuning for clinical data abstraction

To develop an automated pipeline for abstracting structured clinical data, we reviewed previous literature and diagnostic criteria and selected 195 items, including medical history, symptoms, and signs, which were important for the diagnosis of neurodegenerative diseases (Table S1 in supporting information).^9^, ^38^, ^39^, ^40^, ^41^, ^42^ Clinical records sent by a donor's family or from Mayo Clinic's electronic medical records were scanned into PDF format, from which text recognition was performed using PyPDF 2.11.1 and Python‐tesseract 0.3.10. We then searched the document with each search term set for specific symptom and excerpted three lines including it. Patient‐identifiable information was de‐identified; the names of people, facilities, addresses, and dates were redacted using a natural language processing library, spaCy 3.7.2 (ExplosionAI, https://spacy.io).

**FIGURE 3:**
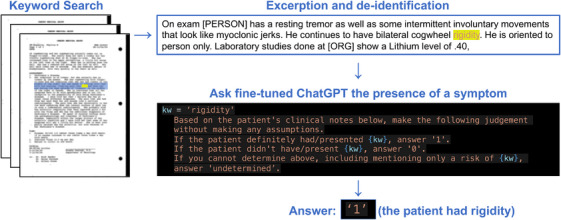
Automated clinical record abstraction. The workflow for automated medical record abstraction is presented. A specific keyword for a symptom is searched for in digitized documents. Three lines containing the keyword are extracted from the matched text and de‐identified. The fine‐tuned ChatGPT is then asked to determine the presence of the symptom. For example, the model is asked whether the patient had rigidity and answers “1” (the patient had rigidity) based on the excerpt.

From the medical records of 504 donors in 2022, 2000 search results with excerpts were randomly selected and annotated by A.R.M. and D.O. The reviewers assessed whether a patient had the symptom, did not have the symptom, or it was not possible to determine, based on an excerpt. A large language model, gpt‐3.5‐turbo‐0613, was fine‐tuned with 1900 human‐labeled excerpts using the ChatGPT API openai 1.33.0. ChatGPT was presented an excerpt and asked to return one out of three options regarding the presence of a symptom. The options were 0 (absent), 1 (present), and “undetermined,” as detailed in Figure 3. To evaluate model performance, accuracy and Cohen's κ were calculated with the fine‐tuned model's responses to the remaining 100 excerpts. To evaluate inter‐rater agreement, the same 100 excerpts were independently annotated by a board‐certified neurologist (H.S.), and those values between human labels were compared.

### 2.6 Summarizing structured clinical data

Structured clinical data obtained by the fine‐tuned GPT were summarized for each patient according to the following workflow: the label “undetermined” was ignored; if a patient had at least one “present” label for a symptom, then the patient was considered to have the symptom; if a patient had any “absent” label without any “present” label, then the patient was considered not to have the symptom; and if a patient did not have any labeling, the patient's value was excluded from relevant analysis as “not available.” To organize the data, key symptoms were grouped in the event that a patient had any of the symptoms in the grouping terms detailed in Table S2 in supporting information. Bradykinesia, in combination with either rigidity or rest tremor, was considered parkinsonism according to the diagnostic criteria.^40^

### 2.7 Statistical analysis

All statistical analyses were performed using scikit‐learn 1.2.0, SciPy 1.9.3, and other related Python libraries. Continuous values were considered non‐parametric and are presented as the median [interquartile range] (number of cases), unless otherwise noted. Statistical differences were assessed using the chi‐squared test for categorical variables and the Mann–Whitney *U* test for continuous variables. Correlations between continuous variables were analyzed using Spearman rank correlation test. A *p* value of < 0.05 was considered statistically significant.

## 3 RESULTS

### 3.1 Patients’ selection and characteristics

In this study, we identified 2702 patients with neuropathologically confirmed AD in the Mayo Clinic Brain Bank, which consisted of 49.0% men, with a median age at death of 80 years [interquartile range: 73, 86] and a median disease duration of 8^6^, ^11^ years. Approximately one third of those with AD (31.3%) had no LB, and no other neurodegenerative diseases (Figure 1 and Table 1).

**TABLE 1:** Comorbid neuropathologies in Alzheimer's disease.

	*n* = 2702
Male, no. (%)	1324 (49.0%)
Age at death (years)	80 [73, 86]
Disease duration (years)	8 [6, 11]
Alzheimer's disease without Lewy bodies	847 (31.3%)
Comorbid pathology	
Lewy body disease	1225 (45.3%)
Cerebrovascular disease	661 (24.5%)
Progressive supranuclear palsy	163 (6.0%)
Frontotemporal lobar degeneration^a^	31 (1.1%)
Corticobasal degeneration	24 (0.9%)
Multiple system atrophy	16 (0.6%)
Amyotrophic lateral sclerosis	10 (0.4%)

*Note*: Data are presented as the number of cases (%) and median [25th, 75th percentiles].

^a^ All patients presented with the TAR DNA‐binding protein 43 subtype.

From this group of AD without LB, we further excluded 78 cases without available midbrain slides and 134 cases with severe artifacts in the SN or no evaluable SN present, resulting in 635 eligible cases for nigral neuron count and clinical record abstraction. Of these cases, 70% had a clinical diagnosis of AD, while others were clinically misdiagnosed with parkinsonian disorders, such as dementia with Lewy bodies (DLB; 11%), CBD (6%), PD (3%), normal pressure hydrocephalus (2%), and PSP (1%; Table 2).

**TABLE 2:** Clinicopathologic characteristics of Alzheimer's disease without Lewy bodies.

		Parkinsonism		
	Overall *n* = 635	Present *n* = 62	Absent *n* = 47	P
Male, no.	295 (46.5%)	35 (56.5%)	25 (53.2%)	0.73
Age at death (years)	78 [70, 85]	80 [71, 84]	74 [68, 86]	0.55
Disease duration (years)	8 [6, 11]	8 [6, 10]	8 [6, 11]	0.97
Brain weight (g)	1040 [940, 1140]	1000 [920, 1120]	1040 [950, 1130]	0.47
Clinical diagnosis^a^				
Alzheimer's disease	447 (70.4%)	35 (56.5%)	31 (66.0%)	0.31
Frontotemporal dementia	76 (12.0%)	5 (8.1%)	6 (12.8%)	0.42
Dementia with Lewy bodies	69 (10.9%)	11 (17.7%)	9 (19.1%)	0.85
Corticobasal syndrome	38 (6.0%)	16 (25.8%)	1 (2.1%)	**<0.01**
Vascular dementia	23 (3.6%)	0 (0.0%)	1 (2.1%)	0.25
Parkinson's disease	18 (2.8%)	9 (14.5%)	1 (2.1%)	**0.03**
Normal pressure hydrocephalus	10 (1.6%)	3 (4.8%)	1 (2.1%)	0.46
Progressive supranuclear palsy syndrome	5 (0.8%)	3 (4.8%)	0 (0.0%)	0.13
*APOE* genotype (*n* = 438)				0.30
ε2/ε3	14 (3.2%)	1 (2.2%)	1 (4.0%)	
ε3/ε3	183 (41.8%)	23 (50.0%)	8 (32.0%)	
ε3/ε4	239 (54.6%)	22 (47.8%)	15 (60.0%)	
ε4/ε4	2 (0.5%)	0 (0.0%)	1 (4.0%)	
*MAPT* genotype (*n* = 231)				0.71
H1H1	143 (61.9%)	11 (57.9%)	8 (72.7%)	
H1H2	69 (29.9%)	5 (26.3%)	2 (18.2%)	
H2H2	19 (8.2%)	3 (15.8%)	1 (9.1%)	
Braak NFT stage	VI [V, VI]	VI [V, VI]	VI [V, VI]	0.27
Thal amyloid phase	5 [5, 5]	5 [5, 5]	5 [5, 5]	0.38
AD subtype				0.95
Typical	338 (65.1%)	30 (56.6%)	19 (54.3%)	
hippocampal sparing	130 (25.0%)	18 (34.0%)	13 (37.1%)	
Limbic	51 (9.8%)	5 (9.4%)	3 (8.6%)	
TDP‐43 in the amygdala	147 (25.0%)	9 (14.8%)	10 (22.7%)	0.3
Nigral neuron density (/mm^2^)	14.9 [11.8, 18.4]	14.1 [11.5, 17.3]	17.0 [14.4, 20.1]	**<0.01**
Pigmented neurons	8.8 [6.6, 11.4]	8.2 [6.6, 10.0]	10.9 [7.6, 12.3]	**<0.01**
Non‐pigmented neurons	6.0 [4.8, 7.2]	6.0 [4.7, 6.8]	6.6 [5.5, 7.9]	**0.03**
Symptoms / signs				
Bradykinesia	74 (67.9%)	62 (100.0%)	12 (25.5%)	**<0.01**
Rigidity	53 (65.4%)	48 (100.0%)	5 (15.2%)	**<0.01**
Rest tremor	21 (46.7%)	14 (48.3%)	7 (43.8%)	0.77
Postural instability	24 (64.9%)	19 (70.4%)	5 (50.0%)	0.25
L‐dopa responsiveness	8 (61.5%)	7 (63.6%)	1 (50.0%)	0.72
Oculomotor dysfunction	28 (32.2%)	24 (45.3%)	4 (11.8%)	**<0.01**
Amnesia	105 (100.0%)	59 (100.0%)	46 (100.0%)	1
Frontal signs	39 (61.9%)	27 (69.2%)	12 (50.0%)	0.13
Cortical signs	38 (77.6%)	25 (86.2%)	13 (65.0%)	0.08
Dream enactment	9 (34.6%)	5 (33.3%)	4 (36.4%)	0.87
Loss of smell	5 (22.7%)	2 (18.2%)	3 (27.3%)	0.61
Visual hallucinations	24 (30.0%)	11 (25.0%)	13 (36.1%)	0.28

*Notes*: Data are presented as the number of cases (%) and median [25th, 75th percentiles]. Statistical differences were evaluated between groups with and without parkinsonism using chi‐square test for categorical variables and the Mann–Whitney *U* test for continuous variables. A value of *p* < 0.05 was considered statistically significant.

Abbreviations: AD, Alzheimer's disease; *APOE*, apolipoprotein E; NFT, neurofibrillary tangle; TDP‐43, TAR DNA‐binding protein 43.

^a^ Some patients have more than one clinical diagnosis.

### 3.2 Nigral neuron count

A machine learning model, YOLOv8, was trained using 3648 tiles from 232 SN with human annotations. The model appropriately detected pigmented and non‐pigmented neurons with a nucleolus with a satisfactory performance of mAP50‐95: 0.651. Neurons on tiled images were remapped to a WSI, and nigral neuron densities were obtained for 635 AD cases without other major neurodegenerative diseases (Figure 2). Pigmented and non‐pigmented nigral neuron densities had a median of 8.8 [6.6, 11.4] /mm^2^ and 6.0 [4.8, 7.2] /mm^2^, respectively (Table 2).

### 3.3 Clinical record abstraction

To efficiently obtain structured clinical information from large datasets, we developed an automated clinical record abstraction pipeline, as shown in Figure 3. We fine‐tuned ChatGPT 3.5 using 1900 human‐labeled excerpts to determine the presence of symptoms. Performance evaluation of the fine‐tuned model on the holdout test dataset showed an accuracy of 82% and a Cohen κ of 0.70, which was an improvement from the pretrained model that had an accuracy of 69% and a Cohen κ of 0.50. The performance of the fine‐tuned model was comparable to the interrater agreement of an independent rater, which had an accuracy of 83% and a Cohen κ of 0.72 (Figure S2 in supporting information). These results ensured the feasibility of the current pipeline for further analysis.

### 3.4 Nigral neuron density and clinical presentation

We abstracted symptoms in 635 cases of AD without LB, of which 109 patients had descriptions of parkinsonian features in their records, and 62 fulfilled the criteria for parkinsonism (bradykinesia and either rigidity or rest tremor; Table 2). There were no significant differences in age at death, disease duration, or apolipoprotein E genotypes between those with and without parkinsonism; however, more patients with parkinsonism were clinically diagnosed with CBS (25.8% vs. 2.1%, *p* < 0.01) and PD (14.5% vs. 2.1%, *p* = 0.03) than those without parkinsonism. The presence of TDP‐43 pathology in the amygdala, which corresponds to LATE, did not differ significantly between these groups. Patients with parkinsonism presented with oculomotor dysfunction more frequently than those without parkinsonism (45.3% vs. 11.8%, *p* < 0.01).

Neuron density in the SN was significantly lower in the patients with parkinsonism compared to those without parkinsonism (Table 3; 14.1 [11.5, 17.3] vs. 17.0 [14.4, 20.1]/mm^2^, *p* < 0.01). In particular, rigidity (14.8 [12.0, 17.7] vs. 17.3 [13.9, 19.4]/mm^2^, *p* < 0.01) was associated with reduced nigral neuron counts. There was no significant correlation between neuron density and rest tremor, postural instability, L‐dopa responsiveness, or other symptoms (Tables 3, S1, and S2).

**TABLE 3:** Nigral neuron densities between cases with and without symptoms.

	Neuron density (/mm^2^)		
Symptoms/signs	Present	Absent	*p*
Parkinsonism	14.1 [11.5, 17.3] (62)	17.0 [14.4, 20.1] (47)	**<0.01**
Bradykinesia	14.5 [11.6, 17.6] (97)	16.2 [14.0, 19.4] (38)	0.07
Rigidity	14.8 [12.0, 17.7] (113)	17.3 [13.9, 19.4] (82)	**<0.01**
Rest tremor	15.8 [13.3, 19.0] (41)	16.7 [13.8, 19.5] (47)	0.60
Postural instability	15.7 [12.4, 18.5] (63)	15.0 [12.6, 17.7] (28)	0.87
L‐dopa responsiveness	15.9 [13.4, 16.3] (10)	14.4 [12.4, 16.0] (8)	0.41
Oculomotor dysfunction	15.2 [13.0, 18.3] (55)	15.6 [12.3, 18.6] (230)	0.51
Amnesia	15.1 [12.0, 18.4] (515)	16.0 [13.8, 17.6] (4)	0.90
Frontal signs	16.1 [13.3, 19.5] (104)	15.1 [12.0, 18.0] (64)	0.20
Cortical signs	15.8 [12.7, 18.6] (111)	16.0 [13.2, 18.0] (42)	0.67
Dream enactment	16.1 [14.5, 17.9] (14)	16.0 [13.3, 18.6] (46)	0.88
Loss of smell	13.5 [11.9, 19.0] (16)	15.8 [13.2, 18.6] (37)	0.95
Visual hallucinations	15.6 [13.1, 19.5] (60)	16.0 [12.7, 18.9] (184)	0.58

*Note*: Data are presented as median [25th, 75th percentiles] (the number of cases evaluated). Statistical differences were evaluated between groups with and without symptoms/signs using the Mann–Whitney *U* test. A value of *p* < 0.05 was considered statistically significant.

### 3.5 Tau immunoreactivity in the substantia nigra

**FIGURE 4:**
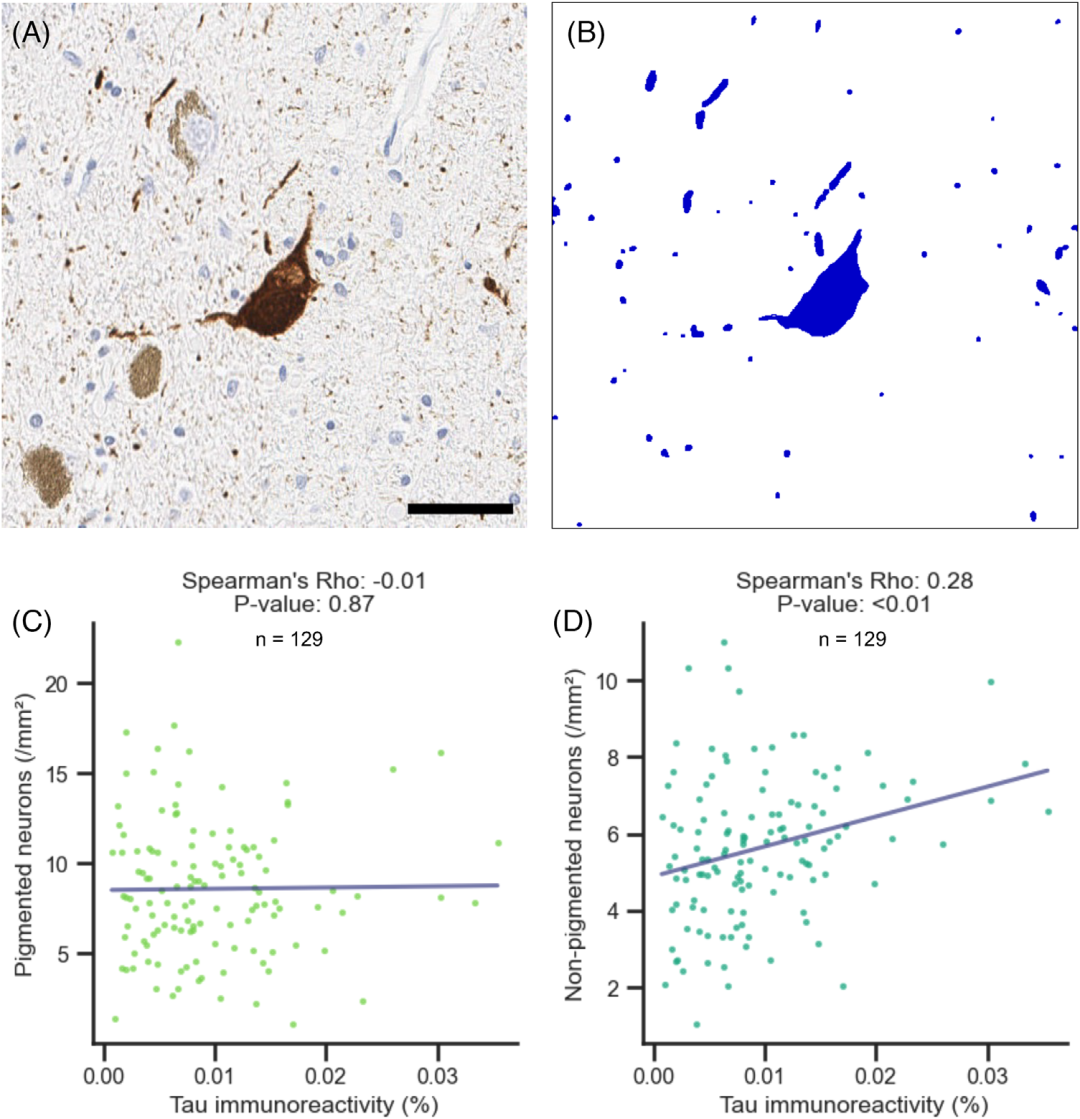
Correlation between nigral tau pathology and neuron density. A, Representative image of tau immunostaining in the substantia nigra. Scale bar: 50 µm. B, Mask image highlighting tau immunoreactive areas (blue). C, Scatter plot showing the correlation between tau immunoreactivity (%) and pigmented neuron density (/mm^2^). Spearman rho: –0.01, *p* value: 0.97. D, Scatter plot showing the correlation between tau immunoreactivity (%) and non‐pigmented neuron density (/mm^2^). Spearman rho: 0.28, *p* value: < 0.01.

Among the 635 AD cases without LB, 130 midbrain sections were randomly selected and evaluated with immunohistochemistry for phosphorylated tau, TDP‐43, and α‐synuclein. One case was excluded from further analysis because α‐synuclein immunostaining revealed a neuron with a Lewy body that was not present in the diagnostic midbrain slide on H&E staining. The remaining 129 cases did not have evidence of α‐synucleinopathy. The Tau immunoreactive area was segmented, and percent occupied area of tau was computed (Figures 4 and S1). There was no significant correlation between tau immunoreactivity and the density of pigmented neurons (Spearman rho: –0.03, *p*: 0.71), whereas a weak positive correlation was found between tau immunoreactivity and the density of non‐pigmented neurons (Spearman rho: 0.24, *p* < 0.01).

### 3.6 Nigral TDP‐43 pathology

**FIGURE 5:**
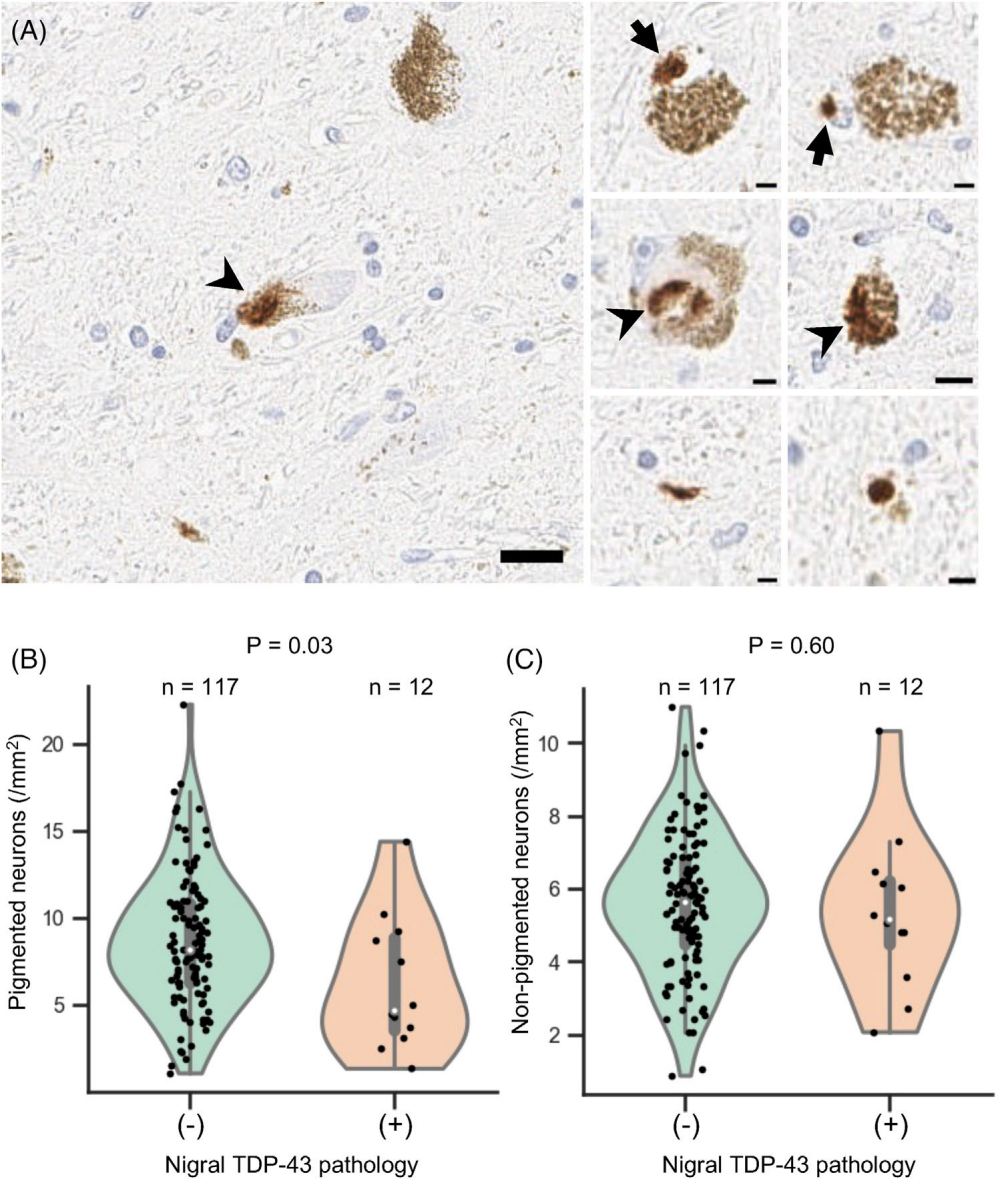
TAR DNA‐binding protein 43 (TDP‐43) pathology and neuron density in the substantia nigra. A, Representative image of TDP‐43 immunostaining in the substantia nigra showing neuronal intranuclear inclusions (arrows), neuronal cytoplasmic inclusions (arrowheads), and extracellular aggregation (right lower panels). Scale bars: 20 µm for the left panel, 5 µm for the right panels. B, Violin plot showing the distribution of pigmented neuron density (/mm^2^) in cases with and without TDP‐43 pathology in the substantia nigra. Mann–Whitney *U* test, *p* = 0.03. C, Violin plot showing the distribution of non‐pigmented neuron density (/mm^2^) in cases with and without TDP‐43 pathology in the substantia nigra. Mann–Whitney *U* test, *p* = 0.60.

Immunohistochemistry for TDP‐43 demonstrated various TDP‐43 pathologies in the SN (Figure 5). Of the 129 slides evaluated, 12 exhibited varying TDP‐43 pathologies such as neuronal cytoplasmic inclusions, neuronal intranuclear inclusions, and extracellular aggregation. There was a significant reduction in pigmented neuronal density in cases with nigral TDP‐43 pathology compared to those without nigral TDP‐43 pathology (4.7 [3.5, 8.9] vs. 8.2 [6.3, 10.8]/mm^2^, *p* = 0.03), whereas no significant difference was observed in non‐pigmented neuron density (5.2 [4.5, 6.2] vs. 5.6 [4.5, 6.8]/mm^2^, *p* = 0.60).

## 4 DISCUSSION

In this study, we investigated clinicopathologic features in AD without LB using data‐driven approach. Our key findings include the following: (1) at least 9.7% of AD patients without LB had parkinsonism, (2) parkinsonism was correlated with reduced nigral neuron density, and (3) TDP‐43 pathology was implicated in nigral neurodegeneration in AD.

Among 635 AD patients without LB, 62 (9.7%) patients presented with parkinsonism. This frequency was lower than the frequencies calculated in the previous clinicopathologic studies (16% and 18%),^1^, ^2^ and is likely due to the limited availability of clinical data in our study. Most patients in this study who had been clinically suspected of having AD were evaluated by dementia specialists or at memory clinics, where motor symptoms may not have been systematically examined. As a result, our study might underestimate the true prevalence of parkinsonism in AD. Nevertheless, given the high prevalence of AD, even this lower frequency of parkinsonism could significantly impact the management of patients with dementia and parkinsonism.

While age‐related parkinsonism has been reported,^43^, ^44^ we did not observe statistically significant differences in age at death between the present patient groups. Patients with parkinsonism were more frequently misdiagnosed with parkinsonian disorders, such as CBD (25.8% vs. 2.1%, *p* < 0.01) and PD (14.5% vs. 2.1%, *p* = 0.03) than those without parkinsonism. No difference between these groups was observed in non‐motor symptoms, such as dream enactment behavior, anosmia, or visual hallucinations, which are characteristic findings in LBD.^40^, ^45^ The patients with parkinsonism tended to have more cortical signs, such as limb apraxia, than those without parkinsonism (86.2% vs. 65.0%, *p* = 0.08, not significant), which corresponds to previous reports on AD‐CBS.^46^, ^47^, ^48^, ^49^ Notably, approximately half of the patients who had parkinsonism had oculomotor dysfunction (45.3% vs. 11.8%, *p* < 0.01), a characteristic feature of PSP. Given that all the patients in this study had amnesia, and predominant impairment of episodic memory is an exclusion criterion for PSP,^42^ most of the patients were not suspected of having PSP. However, three patients (4.8%) in the AD without LB group were clinically diagnosed with PSP based on the presence of parkinsonism and oculomotor dysfunction. To our knowledge, AD with PSP‐like presentation has not been explicitly reported. These findings suggest that AD‐parkinsonism can mimic PD, CBD, and PSP. Biomarkers for amyloid, tau, and α‐synuclein have the potential to aid in differential diagnosis.^9^, ^11^

Our findings suggest that nigral neuronal loss is not associated with high tau burden in the SN but is partially associated with TDP‐43 pathology. We found no correlation between tau immunoreactivity and pigmented neuron density, which is consistent with previous reports that found no association between the nigral tau burden and striatal dopamine transporter expression.^50^ There was a weak positive correlation between tau immunoreactivity and non‐pigmented neuron density (Spearman rho: 0.24, *p* < 0.01). In this study, we used the CP13 antibody, which targets the tau epitope pS202. This antibody primarily recognizes pretangles and tangles, and is less sensitive to ghost tangles that form after neuronal death.^51^ The reduced number of neurons and the progression of NFTs to ghost tangles at advanced stages offers a potential explanation for the mild positive correlation observed in non‐pigmented neurons. Recently, early tau pathology has been reported in elderly individuals (mean age at death: 93 years) with mild parkinsonism, even in the absence of AD or LB pathology.^52^ Further investigation is necessary to determine whether early tau pathology contributes to the degeneration of nigral neurons in AD.

In this study, nigral TDP‐43 pathology, observed in 9.3% of AD without LB, correlated with reduced pigmented neuron density. Previous neuropathologic studies reported that 10% (34/340) of AD patients, including those with comorbid LB, had nigral TDP‐43 pathology, which was aligned with stage 5 in the TDP‑43 in AD staging scheme.^53^, ^54^ Our findings suggest that TDP‐43 pathology, alongside tau pathology, may contribute to pigmented neuron loss in AD without LB.

Our findings are partially consistent with a recent study by Fiondella et al., which demonstrated that nigral TDP‐43 pathology played a significant role in movement disorders among patients with FTLD‐TDP (FTLD with TDP‐43 pathology).^55^ Fiondella et al. observed a higher TDP‐43 burden in the SN among FTLD‐TDP patients with movement disorders compared to those without, despite no significant difference in nigral neuronal density between the groups. These findings support our observation that TDP‐43 pathology, rather than tau burden, is associated with nigral neurodegeneration and the development of parkinsonism in AD without LB. Notably, our study showed a significant difference in SN neuron density between AD patients with and without parkinsonism, highlighting a possible divergence in the pathological mechanisms between AD and FTLD‐TDP. Both Fiondella et al.’s study and our study highlight the importance of considering that TDP‐43–induced neuronal dysfunction may contribute to the development of parkinsonism.

The present study applied a supervised machine learning model for counting neurons on H&E‐stained whole slides, achieving superior performance with a mAP50‐95 of 0.651. Using neuron density obtained from 635 WSIs, we demonstrated the clinicopathologic association of nigral neuron loss and parkinsonism in AD without LB, which has not been previously reported. This automated method for whole slide neuron count will be expanded to other brain regions for more accurate and interpretable measurement.

We implemented a pipeline to automatically abstract structured clinical data from medical records. Previous studies have highlighted the challenges of applying large language models, particularly in terms of reliability in complex tasks and interpretability of their predictions.^24^, ^25^, ^26^, ^27^, ^28^, ^29^ The current study aimed to address these limitations by dividing the tasks of abstracting symptom into three simpler tasks: keyword search, extraction and de‐identification, and ChatGPT query. The first two tasks are reliable because they are rule based. The final step showed improved accuracy through fine‐tuning the model with human labels. Mislabeling by the fine‐tuned ChatGPT was observed only for labels marked as “undetermined,” where human annotated “present” or “absent,” but ChatGPT predicted “undetermined,” or vice versa. These excerpts often lacked sufficient information necessary for a human rater to make a definitive determination of symptom presence, as evidenced by the similar inter‐rater agreement and model performance (accuracy: 83% vs. 82%, Figure S2). Overall, the pipeline successfully automates the abstraction of structured clinical data, enabling detailed phenotyping with large datasets.

Recent clinical trials have demonstrated the effectiveness of anti‐amyloid therapy for early AD.^10^, ^12^, ^13^ These trials excluded candidates who were determined to have causes of dementia other than AD. As dementia with parkinsonism meets the clinical diagnostic criteria for possible DLB, it is likely that AD patients with parkinsonism were excluded from these trials.^45^ However, it is reasonable to assume that anti‐amyloid therapy will alleviate the progression of dementia caused by AD pathology, even for patients with parkinsonism. To avoid missing potential opportunities for patients to benefit from anti‐amyloid therapy, it is necessary to recognize the clinical significance of parkinsonism in AD without LB.

This present retrospective study, which primarily focused on the analysis of the large number of clinicopathologic datasets, has the following limitations. As previously discussed, the availability of the records was limited, and available records lacked standardized structures or clinical descriptions. In addition, the relationship between nigral TDP‐43 pathology and parkinsonism remains to be investigated with a larger number of cases with recorded parkinsonism status. Our study abstracted the lifetime presence of symptoms, but did not assess the onset and severity of those symptoms. The addition of data pertaining to the severity and temporal progression of parkinsonism would provide a more detailed clinical picture of AD with parkinsonism. We also acknowledge that our analysis, which evaluated nigral neuronal density from a single standardized section, does not account for the total SN neuronal count as a three‐dimensional structure. While stereological validation could provide further insights, it is beyond the scope of this study. The current method analyzed all regions of the SN on a midbrain section, including the pars reticulata and pars compacta. Region‐specific neuronal loss in SN subregions remains to be investigated in AD without LB.

In conclusion, this study demonstrated that a subset of those with AD present with parkinsonism and nigral neuronal loss, which is not associated with LB. This finding has important implications in the era of disease‐modifying therapy to ensure that potential anti‐amyloid treatment opportunities are not missed.

## CONFLICT OF INTEREST STATEMENT

The authors have no competing interests to declare. Author disclosures are available in the supporting information.

## CONSENT STATEMENT

Brain autopsies were performed with the consent of legal next of kin or individuals with legal authority to grant permission for autopsy. This study was approved by the Mayo Clinic Institutional Review Board (24‐006694).

## Supporting information



Supporting Information

Supporting Information

Supporting Information

Supporting Information
