# Supplementary material for: Parkinsonism in Alzheimer's disease without Lewy bodies in association with nigral neuron loss: A data‐driven clinicopathologic study
Source: Alzheimers Dement. 2025 Mar 5;21(3):e14628. doi: 10.1002/alz.14628 (PMC11881629; doi:10.1002/alz.14628)
Supplement: Supplementary file 1 — Supporting Information [file ALZ-21-e14628-s001.docx]

**FIGURE S1 Segmentation of tau immunoreactive area**

(A) Representative image of the substantia nigra showing tau immunoreactivity. Scale bar: 50 µm. (B) Workflow for segmenting tau-immunoreactive areas: tau-immunoreactive objects and pigments are initially segmented using a low-intensity threshold on the DAB channel (intensity > 0.3, gray). Objects exceeding this threshold and larger than 50 µm² (green), including pigments, are then subtracted. High-intensity objects (intensity > 0.8, red), such as neurofibrillary tangles, are identified and added to the subtracted mask. The final segmentation isolates tau-immunoreactive areas while excluding pigments (blue).

**FIGURE** **S2 Performances of GPT’s prediction and interrater agreement**

(A) Confusion matrix comparing the predictions of the pre-trained GPT model with the annotations of Rater 1. The pre-trained GPT model has an accuracy of 69% and a Cohen's κ of 0.50. (B) Confusion matrix comparing the predictions of the fine-tuned GPT model with Rater 1 annotations. The fine-tuned GPT model achieved an accuracy of 82% and a Cohen's κ of 0.70. (C) Confusion matrix comparing Rater 1 and Rater 2 annotations for interrater agreement. The interrater agreement showed an accuracy of 83% and a Cohen's κ of 0.72.

**TABLE S1 Presence of symptoms and nigral neuron density**

Data are presented as the number of cases (%) and median [25^th^, 75^th^ percentiles]. Statistical differences were evaluated using chi-square test for categorical values and using the Mann-Whitney U test for continuous values. A value of P < 0.05 was considered statistically significant.

**TABLE S2 Presence of symptom groups and nigral neuron density**

Data are presented as the number of cases (%) and median [25^th^, 75^th^ percentiles]. Statistical differences were evaluated using chi-square test for categorical values and using the Mann-Whitney U test for continuous values. A value of P < 0.05 was considered statistically significant.
